# Follicular adenoma with bizarre nuclei and wild-type P53 expression: A case report and literature review

**DOI:** 10.1177/20363613231212383

**Published:** 2023-10-28

**Authors:** Daniel Nguyen, Nyein Nyein Htun, Beverly Wang

**Affiliations:** Department of Pathology and Laboratory Medicine, 14447 University of California at Irvine Health System, Orange, CA, USA

**Keywords:** Follicular thyroid adenoma, bizzare nuclei, p53 immunohistochemistry, thyroid nodules, thyroidectomy

## Abstract

**Introduction:**

Thyroid cancer is the most common endocrine tumor in humans. Follicular adenoma/carcinoma is the second most common subtype. Multiple histological patterns have been identified. Follicular adenoma with bizarre nuclei is one of the patterns associated with p53 mutation and has an unclear clinical prognosis.

**Case report:**

A 74-year-old female presented with incidental findings of elevated TSH levels and normal thyroid markers. Ultrasound was performed and revealed multiple bilateral thyroid nodules measuring up to 1.9 cm. Fine needle aspiration was performed, and cytology showed one Bethesda category 5 nodule. Total thyroidectomy with neck dissection was performed, and the pathology showed follicular adenoma with bizarre nuclei. Based on the results of immunohistochemistry, the neoplastic cells exhibited staining for wild-type p53 and low levels of the proliferation index Ki-67.

**Conclusions:**

We report a rare case of thyroid follicular adenoma with bizarre nuclei. In contrast to previous reports of this tumor, our patient showed a p53 wild-type pattern using immunohistochemistry. More studies are needed to better understand the etiology and clinical prognosis of this tumor.

## Introduction

Thyroid cancer is the most common endocrine tumor in humans and increasing incidence has been reported.^1^ The most common subtype is papillary thyroid carcinoma.^2^ Follicular adenoma (FA) and follicular thyroid carcinoma (FTC) are neoplasms consisting of differentiated follicular cells.^3^ Follicular adenoma with bizarre nuclei is a rare tumor subtype of follicular adenoma. According to previous reports, it is associated with p53 mutation.^4,5^

Here, we report a case of a 74-year-old female with follicular adenoma with bizarre nuclei and wild-type p53 immunohistochemical staining.

## Case report

**Figure 1.**
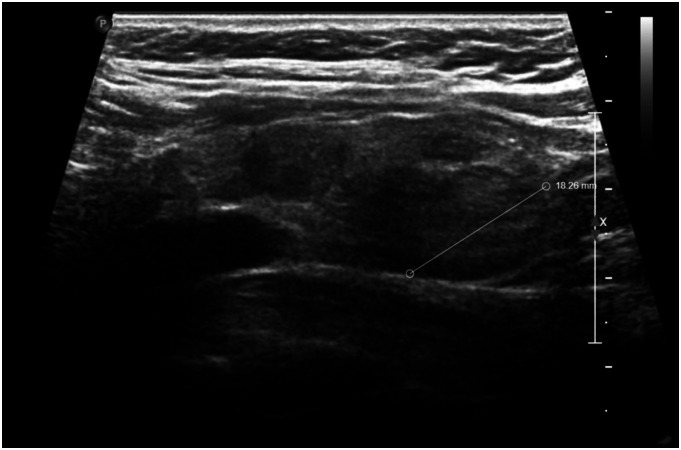
Thyroid ultrasound shows multiple thyroid nodules.

A 74-year-old female patient presented with a past medical history of dementia, anemia, liver disease, and thrombocytopenia. Two years ago, during a routine laboratory workup with her primary care physician outside our institution, her TSH level was elevated to 5.79 mIU/L (normal 0.40–4.50 mIU/L) with normal free T4 and total T3 levels and she was negative for thyroglobulin and thyroid peroxidase antibodies. No medication was prescribed, and the patient was closely followed. One year later, her TSH level remained elevated at 5.24 mIU/L, and thus her primary care physician ordered an ultrasound thyroid that showed multiple bilateral hypoechoic nodules measuring up to 1.9 cm (Figure 1). The findings prompted the clinician to perform a fine needle aspiration biopsy; a Bethesda Category 5 nodule indicating a suspicion for malignancy was detected in the right superior lobe of the thyroid gland. The patient was referred to our hospital for further work-up and a higher level of care.

The patient was evaluated by our otolaryngology surgery department. She had no compressive symptoms, hoarseness, dysphagia, hemoptysis, otalgia, shortness of breath, or weight loss. Upon palpation, multiple thyroid nodules were detected, which were confirmed by an in-house ultrasound. The results from the remaining exams were unremarkable. The consensus between the patient and the clinician was to perform a total thyroidectomy. The patient underwent total thyroidectomy approximately 6 months after her diagnosis of a Bethesda Category 5 nodule.

**Figure 2.**
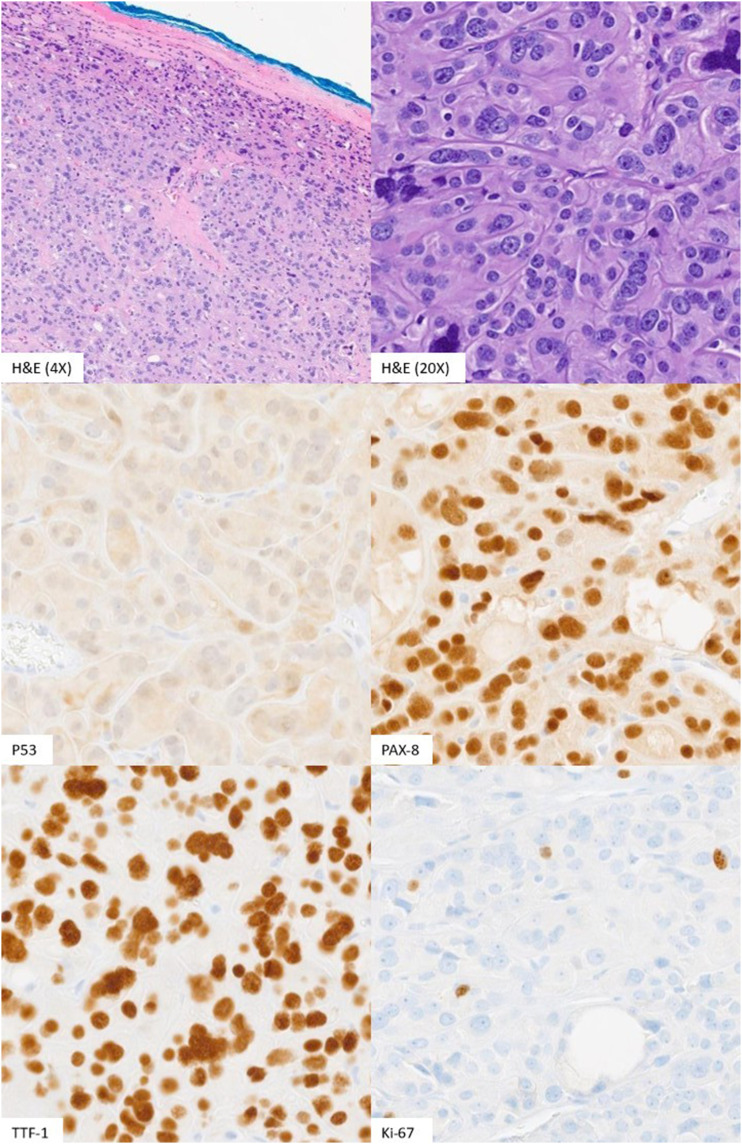
H&E and immunohistochemical staining of the tumor.

Our pathology department received total thyroidectomy specimens after central neck dissection. Macroscopically, multiple tan, focally hemorrhagic, irregular nodules ranging from 0.3 to 1.5 cm were observed throughout the left and right lobes. Microscopically, we observed an encapsulated follicular adenomatous neoplasm with a trabecular pattern and hyalinizing stroma (PAS staining). Numerous bizarre nuclei with significant pleomorphism were observed. Bizarre multinucleated giant cells were present with prominent nucleoli. No lymphovascular or capsular invasion was observed. The neoplastic cells were positive for PAX-8, TTF-1, and Vimentin and focally positive for synaptophysin. The neoplastic cells were negative for chromogranin and BRAF V600E. The expression pattern of p53 was wild-type with a low level of the proliferation index Ki-67 (Figure 2). The histomorphology of the tumor was consistent with the entity named follicular adenoma with bizarre nuclei. We sent the case to another institution for a second opinion, and the expert agreed with our diagnosis.

The patient recovered well from the surgery with no complications and received regular follow up with the surgeon. Her TSH level returned to normal. No further treatment was recommended by the clinical team.

## Discussion

Thyroid cancer is the most common endocrine cancer in humans. Data show that it is more common in females than in males (3:1) and has shown increasing trends recently, irrespective of geography.^1^ The mean age at diagnosis is 48 years.^6^ The main subtypes of thyroid cancer include papillary thyroid cancer, follicular thyroid cancer, medullary thyroid cancer, and anaplastic thyroid cancer, with papillary cancer identified as the most common type.^2^ Some of the risk factors for thyroid cancer include exposure to radiation and a patient or family history of benign thyroid disease, such as multiglandular disease and hyperthyroidism and malignant thyroid cancer.^7^

Follicular adenoma and follicular carcinoma of the thyroid gland are tumors of the follicular cells. Follicular adenomas are benign neoplasms with an incidence of 2%–4.3% of the population. Follicular thyroid neoplasms share many clinical and cytological characteristics, and it is difficult to differentiate between benign and malignant thyroid nodules.^3^ Clinical symptoms include thyroid nodules, a history of thyroid autoimmune disease or, rarely, pain and dyspnea.^8^ Calcification, tubercle in nodule signs, spiculated margins and trabecular formations are more prevalent in follicular carcinoma, facilitating the differentiation between follicular adenoma and carcinoma.^3^ Follicular carcinoma is distinguished from follicular adenoma by its invasion outside of the capsule or metastasis to lymph nodes or other organs.^9^ The Bethesda System for Reporting Thyroid Cytopathology, 2017 revision, includes six diagnostic categories. The fine needle aspiration biopsy from this patient revealed a category 5 nodule, which has a 45%–60% risk of malignancy, and the eye-catching nature of bizarre nuclei prompted to a near-total thyroidectomy or lobectomy.^10^ Due to the rarity of the tumor in literature, no specific cytological findings have been characterized. In one reported follicular adenoma with bizarre nuclei case, fine needle aspiration was performed, and the cytology finding was suspicious for papillary thyroid carcinoma which is similar to the report in our case.^5^ In our case, using Automated Intelligence (AI) and Whole-Slide Scanning may be useful to evaluate the malignant potential of the tumor in needle aspiration. By defining and comparing cytological and possible immunohistochemical features between follicular adenoma with bizarre nuclei to malignant thyroid carcinoma, it may increase the accuracy of fine needle aspiration and prevent the patient from underwent unnecessary procedure.^11^

Based on the 2022 Endocrine and Neuroendocrine WHO Classifications of Tumours, the diagnosis of our patient is most consistent with follicular thyroid adenoma with bizarre nuclei and histological patterns. Despite being swayed by the presence of neoplastic cells with enlarged and hyperchromatic nuclei, the absence of mitotic activity and tumor necrosis endorse the nature of follicular adenoma with bizarre nuclei.^12^ No evidence of necrosis along with very low mitotic activity is observed with a multilevel examination. No macroscopic or microscopic evidence of capsular invasion, lymphovascular invasion, or any other metastasis to lymph nodes or other organs reassured the benign nature of this tumor. Using immunohistochemical staining, p53 exhibited the wild-type expression pattern.

From our literature review, p53 is expressed with mutant patterns in follicular thyroid adenoma with bizarre nuclei. It is reported to strongly and diffusely stained bizarre nuclei but not normal follicular cells.^4,5^ P53 is often expressed aberrantly in the more aggressive and undifferentiated thyroid carcinoma.^13^ In our case, p53-positive staining was observed in a few of the bizarre neoplastic cells, consistent with wild-type expression.

## Conclusion

In conclusion, we reported a case of follicular thyroid adenoma with bizarre nuclei presenting wild type p53 expression. More studies are needed to fully understand the tumorigenesis, clinical behavior and specific histopathological features of this rare type of tumor.

## Appendix

### Abbreviations

P53: Tumor Protein P53
TSH: Thyroid Stimulating Hormone
PAS: Periodic acid–Schiff
PAX-8: Paired box gene 8
TTF-1: Thyroid transcription factor-1
BRAF: Serine/threonine-protein kinase B-Raf.
